# Supplementary material for: Planar photonic chips with tailored angular transmission for high-contrast-imaging devices
Source: Nat Commun. 2021 Nov 25;12:6835. doi: 10.1038/s41467-021-27231-6 (PMC8616932; doi:10.1038/s41467-021-27231-6)
Supplement: Supplementary file 1 — Suplementary information [file 41467_2021_27231_MOESM1_ESM.pdf]

SUPPLEMENTARY INFORMATION

Planar photonic chips with tailored angular transmission for high-contrast-imaging devices

Yan Kuai[1], Junxue Chen[2], Zetao Fan[1], Gang Zou[3], Joseph. R. Lakowicz[4], and Douguo Zhang[1]†

[1]Hefei National Laboratory for Physical Sciences at the Microscale, Advanced Laser Technology Laboratory of Anhui Province, Department of Optics and Optical Engineering, University of Science and Technology of China, Hefei, Anhui, 230026, China

[2]College of Science, Guilin University of Technology, Guilin, Guangxi, 541004, China

[3]CAS Key Laboratory of Soft Matter Chemistry, Department of Polymer Science and Engineering, University of Science and Technology of China, 230026 Hefei, Anhui, China

[4]Center for Fluorescence Spectroscopy, Department of Biochemistry and Molecular Biology University of Maryland School of Medicine, Baltimore, Maryland 21201, USA

†Correspondence and requests for materials should be addressed to: dgzhang@ustc.edu.cn (Douguo Zhang)

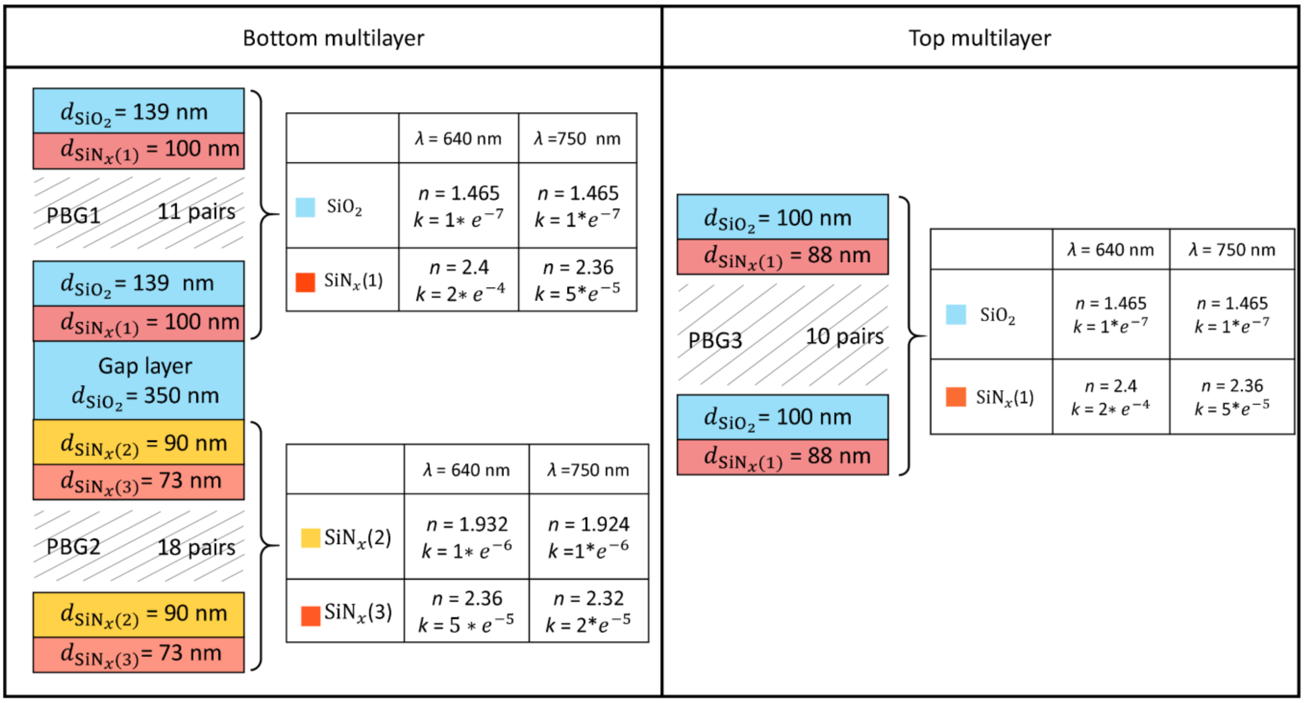

Figure S1. Structural parameters of the top and bottom multilayer.

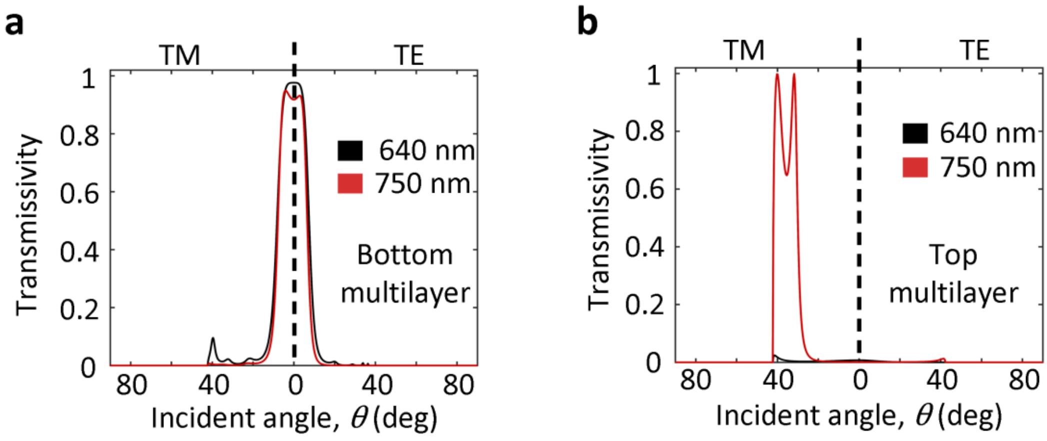

Figure S2. Angular dependent transmittivity of the bottom and top multilayer. (a) the bottom multilayer; (b) the top multilayer. The incident wavelength is 640 and 740 nm. The incident polarization is TM (left) or TE (right).

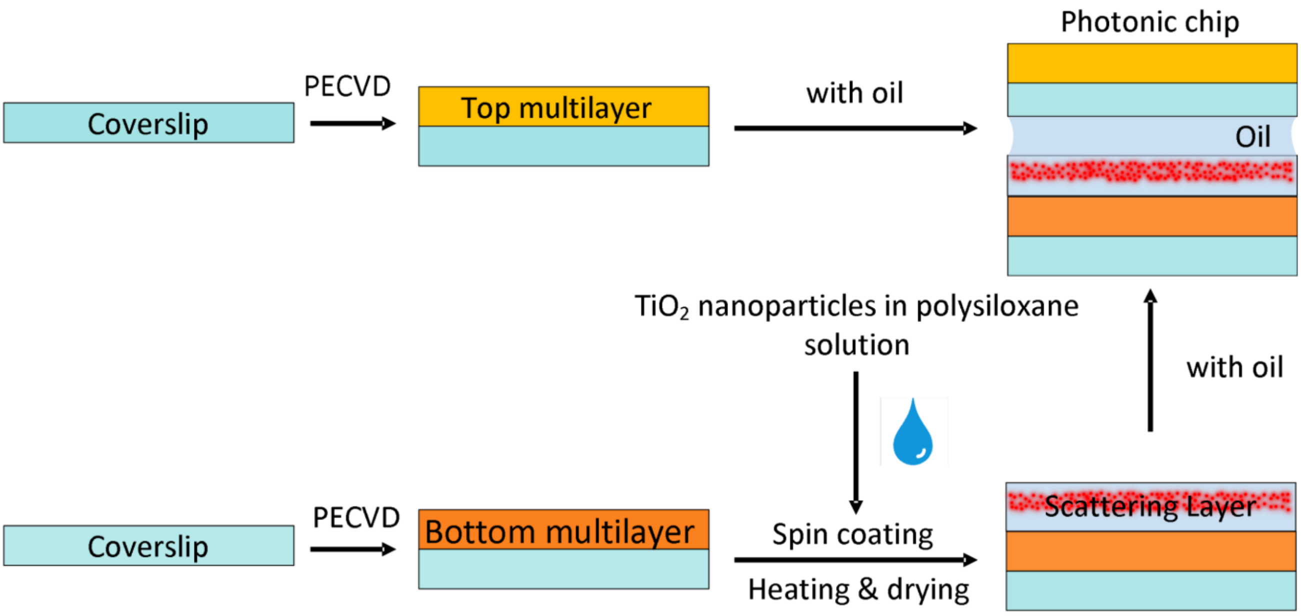

Figure S3. Schematic of the manufacturing procedure for the proposed photonic chip

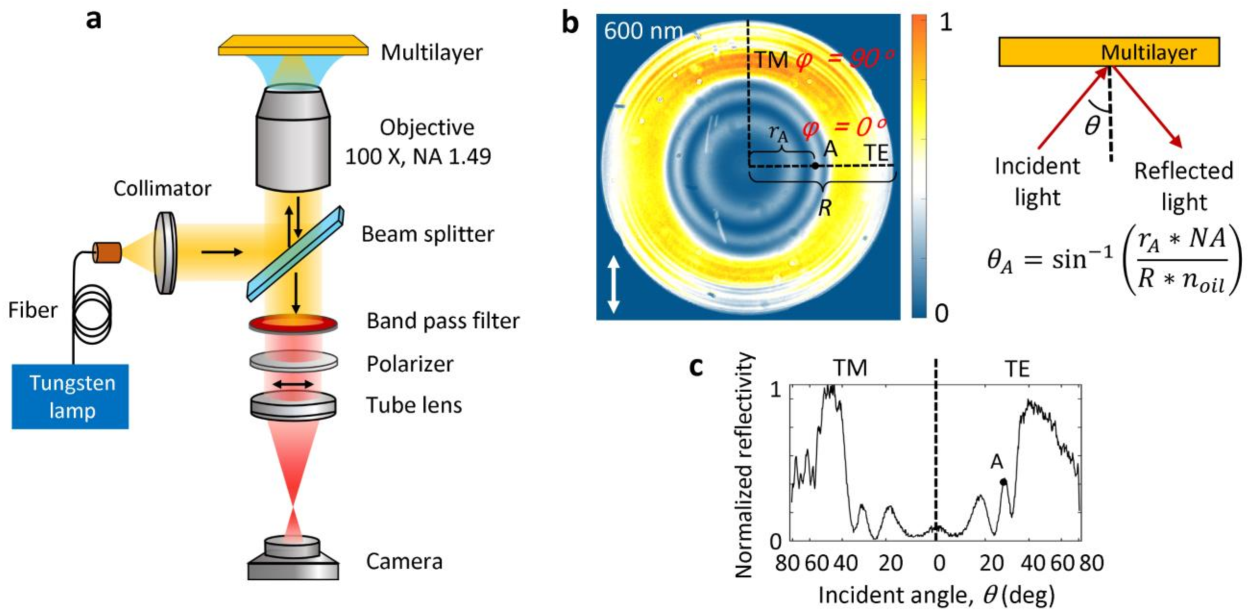

Figure S4. Schematic of the experimental setup for measuring the reflection BFP images from the top and bottom multilayer. (a) An expanded white light beam (from Tungsten lamp) is focused on the the dielectric multilayer and the reflected light is collected using the same objective. The BFP of the objective is imaged using the sCMOS. A serial of band-pass filters with center wavelengths ranging from 600 nm to 790 nm was placed before the sCMOS to filter the reflected light. A polarizer is placed before the tube lens to check the polarization states of the reflected light. (b)The BFP image of the top multilayer with incident wavelength at 640 nm. The arrow line represents the orientation of the polarizer. Every spot on the BFP image represents the information on an angle (polar angle θ and azimuthally angle *φ*). The center point of the BFP image is the origin where the θ = φ = 0^0. The polar angle related with spot A can be derived from the known N.A of the objective and the radius (*r*, and *R*). The azimuthally angle was defined according to the orientation of the polarizer. The horizontal direction (indicated with the dashed line) corresponds to φ = 0^0, then the reflected light on this dashed-line is of TM- polarization relative to the surface of the multilayer. The reflected light on the direction (φ = 90^0) is of TE-polarization. (c) Intensity profiles extracted along the black dashed lines

on (b) can be used to derive the angular dependent reflectivity for both TM and TE-polarized incident beam at the 640 nm wavelength.

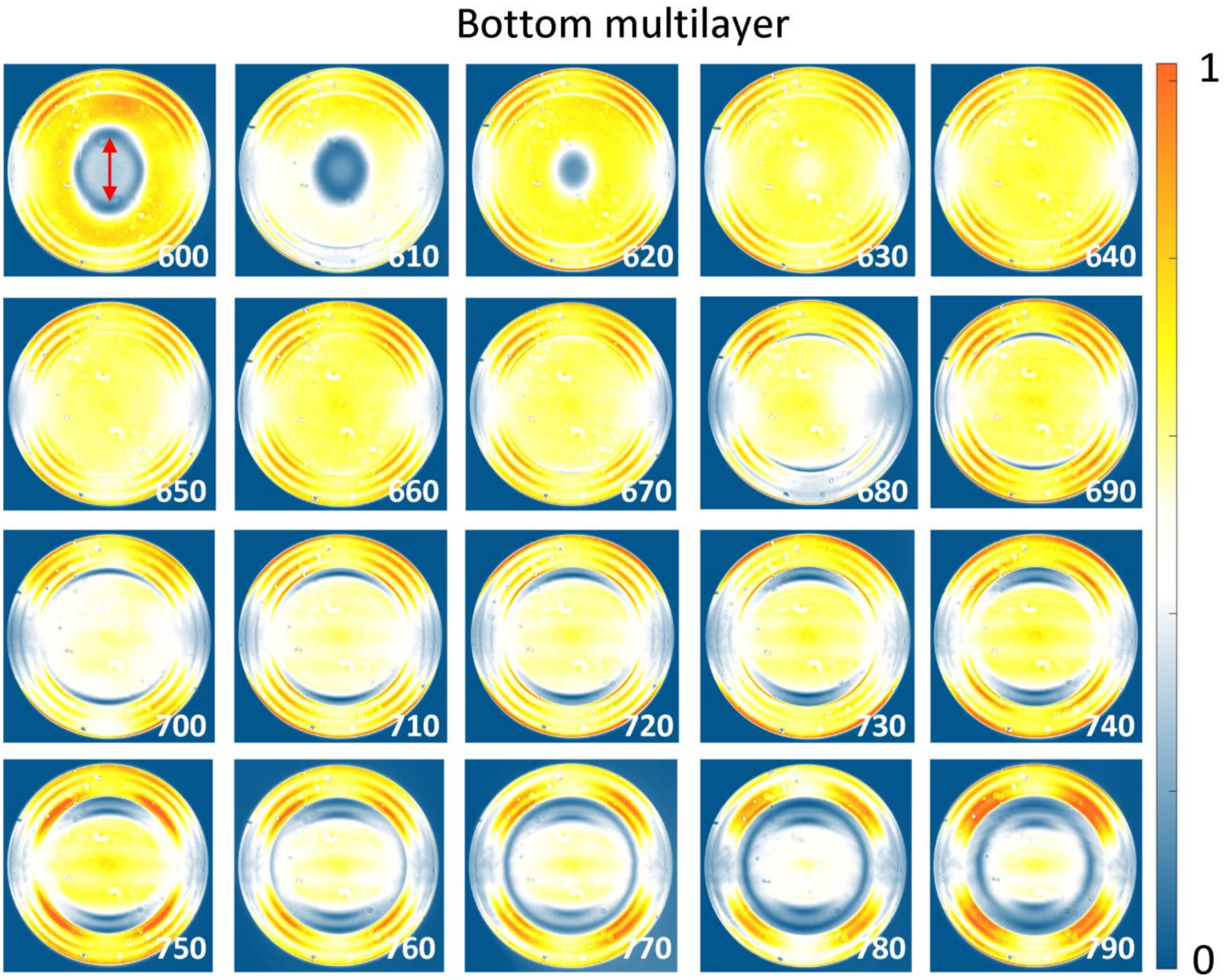

Figure S5. Reflection BFP images of the bottom multilayer with incident wavelengths ranging from 600 nm to 790 nm. The arrow line on the first image (incident wavelength = 600 nm) represents the orientation of the polarizer. The angular dependent reflectivity for both TM and TE-polarized incident beam at each wavelength can be derived from each BFP image, by using the procedures shown in Figure S4. Then, the PBGs of the bottom multilayer can be obtained as shown in Figure 2c.

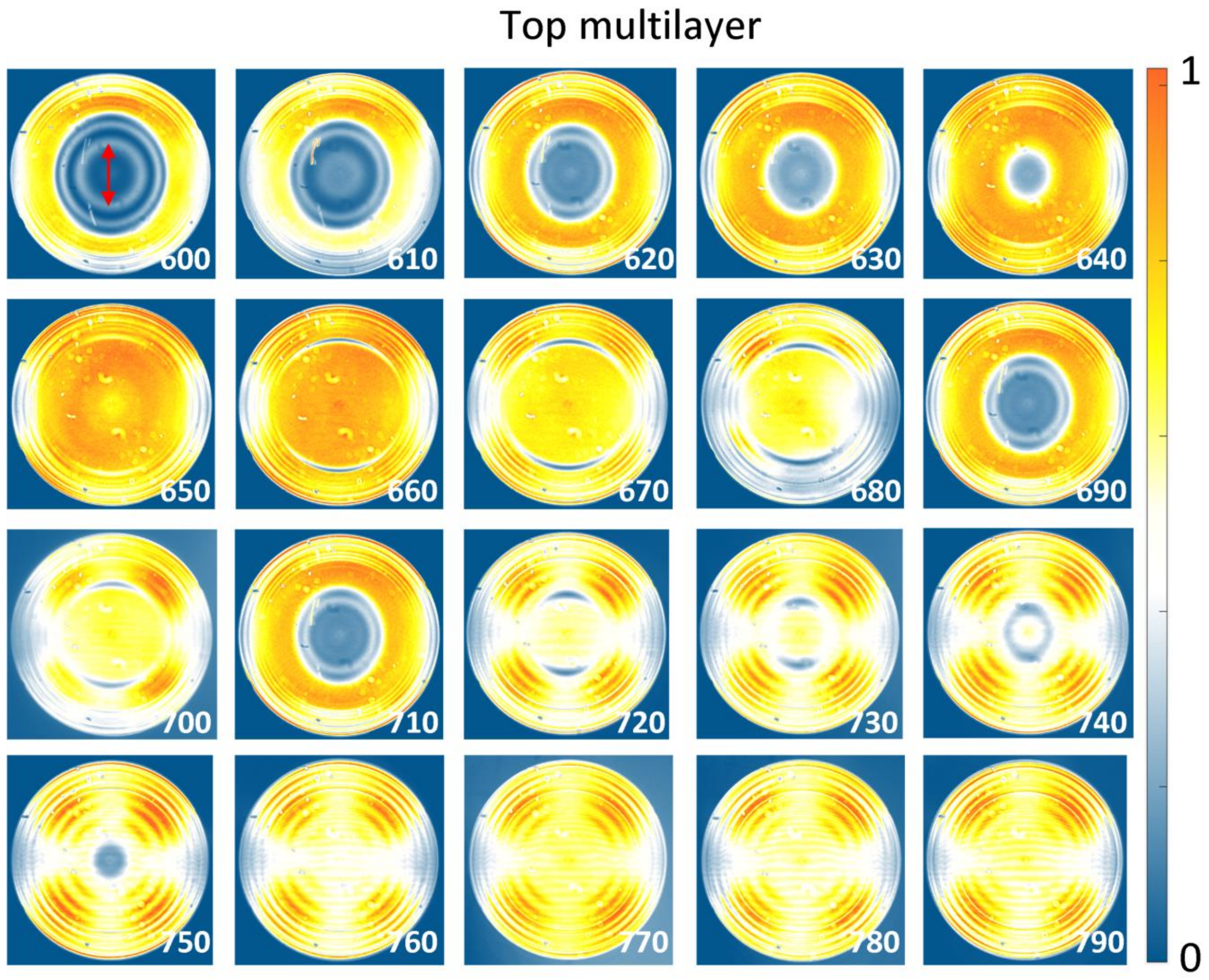

Figure S6. Reflection BFP images of the top multilayer with incident wavelengths ranging from 600 nm to 790 nm. The arrow line on the first image (incident wavelength = 600 nm) represents the orientation of the polarizer. The angular dependent reflectivity for both TM and TE-polarized incident beam at each wavelength can be derived from each BFP image, by using the procedures shown in Figure S4. Then, the PBGs of the top multilayer can be obtained as shown in Figure 2d.

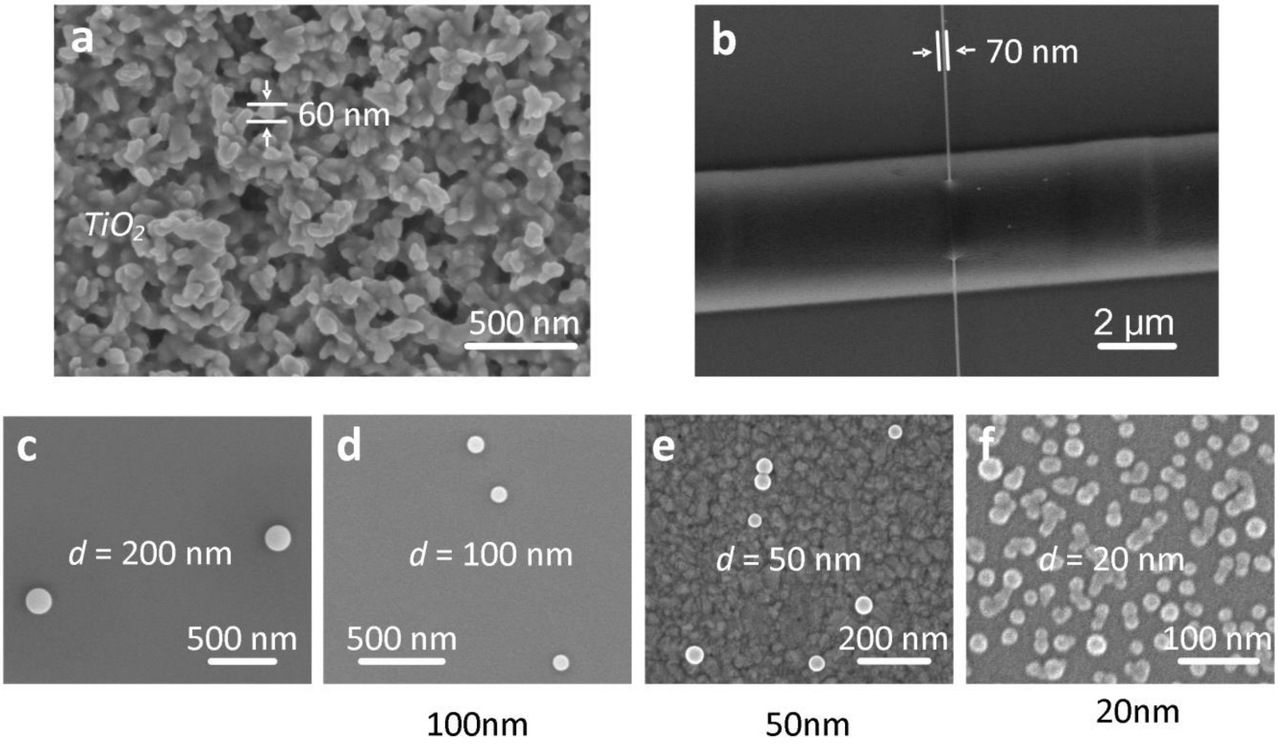

Figure S7. Scanning electron microscope images of the TiO2 nanoparticles (a), the polymer microwire and nanowire (b), the polystyrene nanoparticles with diameter at 200 nm (c), 100 nm (d), 50 nm (e), and 20 nm (f).

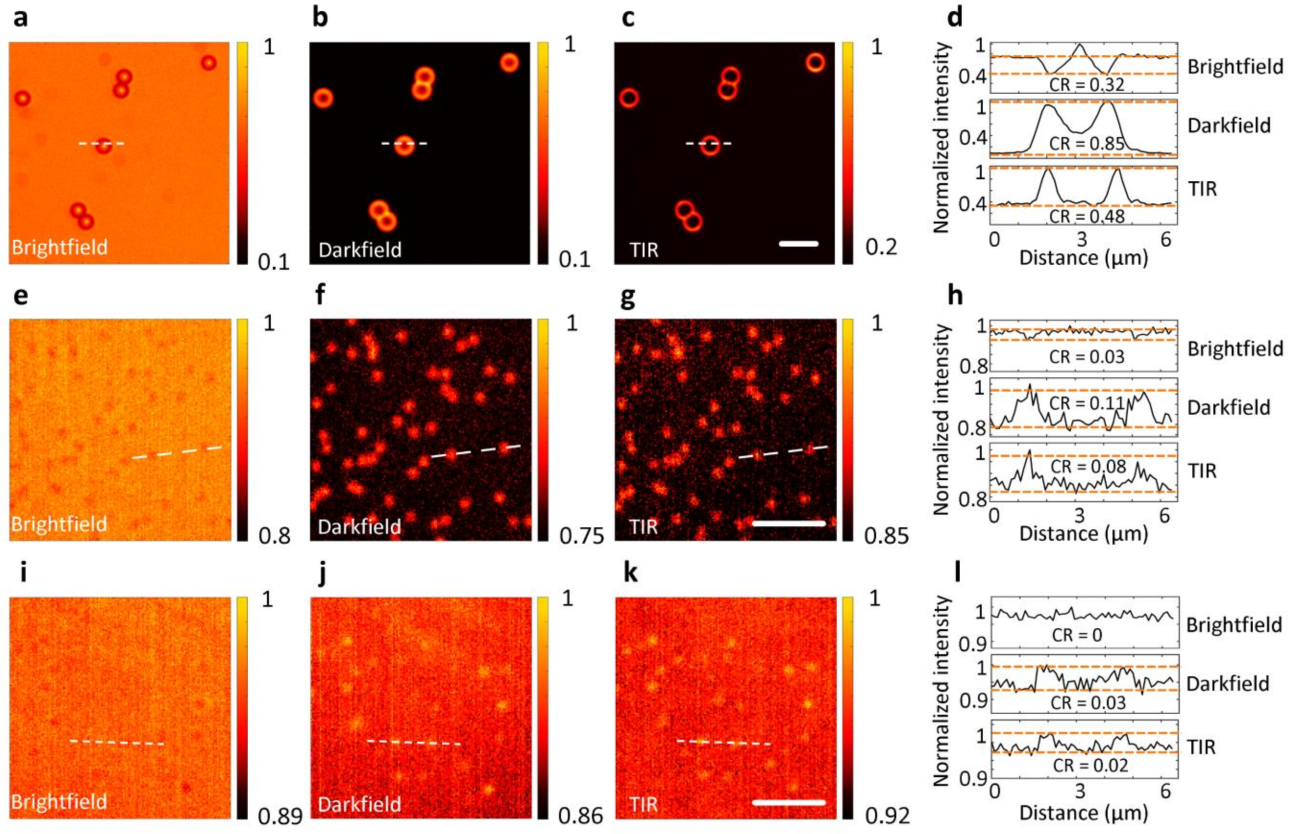

Figure S8. Brightfield, darkfield and TIR images of the polymer particles. The diameter of the particles is 2 μm (a-d), 200 nm (e-h), and 20 nm (i-l). (d) Intensity profiles extracted along the white dashed lines on the three kinds of images. The dashed red lines indicate the levels used to determine the image contrasts (CR). Scale bars, 5 μm. For the brightfield images, the particles were placed on a bare coverslip. For the darkfield (750 nm incident wavelength) and TIR (640 nm incident wavelength) images, the photonic chip was attached below this coverslip. The contrast of the darkfield and TIR images are better than that of the brightfield ones for all these polymer particles. The sharper edges of the polymer particles on (c) than that on (b), meaning the higher spatial resolution of the TIR imaging than that of the darkfield imaging. The images were captured with the standard microscopy shown in Figure 4(a) with a regular air objective (60X, NA = 0.7).

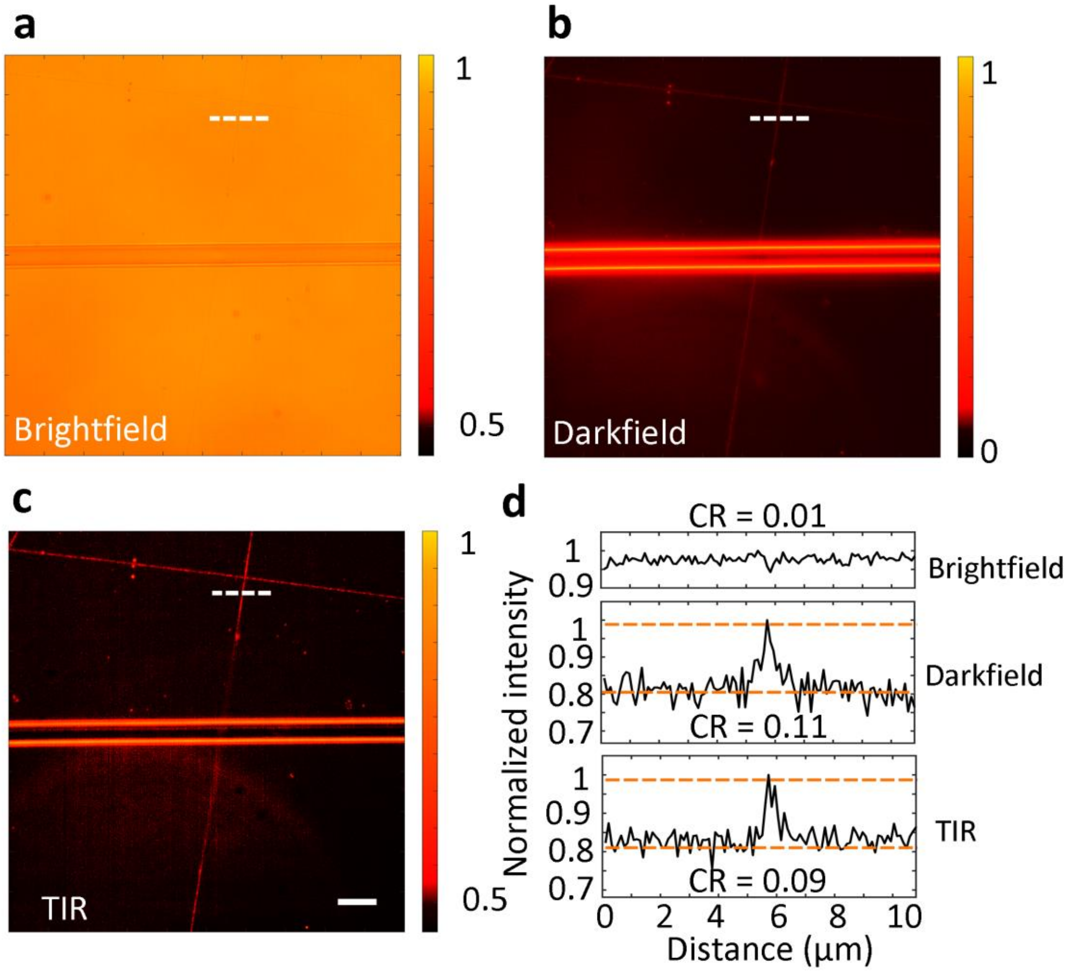

Figure S9. Brightfield (a), darkfield (b) and TIR (c) images of the polymer nanowires and microwires immersed in water solution. (d) Intensity profiles extracted along the white dashed lines on (a, b and c). The dashed red lines indicate the levels used to determine the image contrasts (CR). Scale bars 20 µm. The images were captured with the standard microscopy shown in Figure 4(a) with a regular air objective (40X, NA = 0.6).

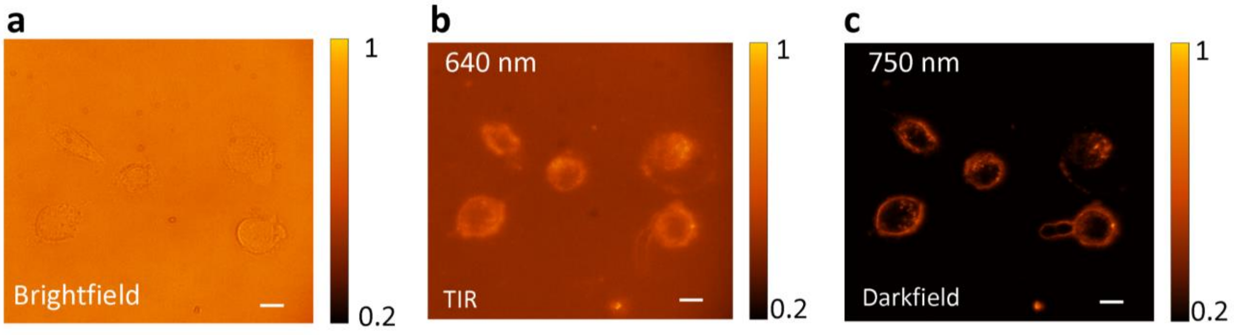

Figure S10. Brightfield (a), TIR (b) and darkfield (c) images of the biological cells (CT26, a murine colorectal carcinoma cell line which is from a BALB/c mouse) cultured in Dulbecco's Modified Eagle Medium (DMEM) with 10% serum. The images were captured with the standard microscopy shown in Figure 4(a) with a regular air objective (40X, NA = 0.6). Scale bars 10 µm.

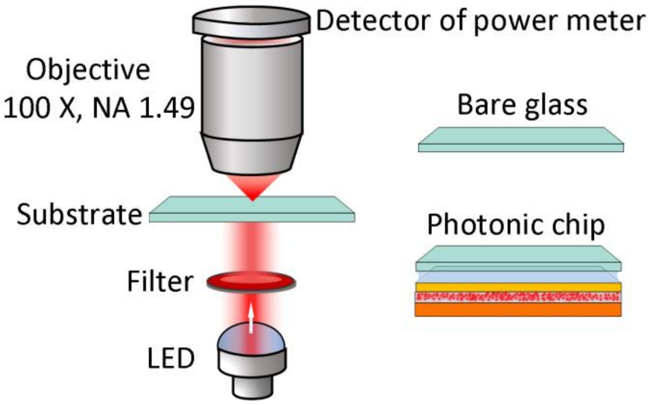

Figure S11. Experimental setup for measuring the coupling efficiency of the photonic chip. Two control substrates were used. One is the bare glass substrate, and the other is the photonic chip. A power meter was used to measure the transmitted power through the two substrates.
